# Sustainable Lifestyle Change—Participatory Design of Support Together with Persons with Obesity in the Third Age

**DOI:** 10.3390/ijerph13121248

**Published:** 2016-12-16

**Authors:** Sarianne Wiklund Axelsson, Åsa Wikberg-Nilsson, Anita Melander Wikman

**Affiliations:** 1 Division of Health and Rehabilitation, Department of Health Sciences, Luleå University of Technology, Luleå 97187, Sweden; 2 Division of Innovation and Design, Department of Business Administration, Technology and Social Sciences, Luleå University of Technology, Luleå 97187, Sweden; Asa.wikberg-nilsson@ltu.se

**Keywords:** older persons, life-style change, m-health, participatory design

## Abstract

Sustainable lifestyle changes due to obesity are difficult to achieve regardless methods used. We need to know more about the lived experience of obesity and older persons’ needs for support to make a sustainable change. This paper focuses on the need-finding process in designing support for a sustainable lifestyle change. Multistage focus group interviews were conducted with persons aged 61–72 living in Northern Sweden. A participatory and appreciative reflection and action (PAAR) approach was used in the group-sessions. Probes were used to increase reflections and achieve a deeper knowledge about the participants’ needs of support. Data were analysed using qualitative thematic content analysis. Our findings revealed that to be able to succeed with a lifestyle change a focus has to be on a converted way of thinking, managing vulnerability, and achieving an emotional balance. To achieve a sustainable lifestyle change due to obesity in the third age the focus has to be on a health identity instead of a weight identity. Personalised support with enjoyable physical activities should be designed and developed. Strategies for emotional balance based on autonomy and self-empowerment must be included. This knowledge is important when designing support for sustainable change.

## 1. Introduction

In order to design and tailor personalised health-related applications that support sustainable lifestyle changes, we have to understand the explicit needs among the special target group. Deep understanding is based on a wide range of psychological mechanisms that include situations and feelings [1]. People have the right to influence the system that they will use, and, therefore, they should have a “voice” and an ability to exert influence throughout the process. Design with users is an approach whereby products and services are co-designed by developers and users. A conceptualisation of user needs is a prerequisite for development of systems in information technology (IT) design [2]. Need finding is the foundation for recruitment of specifications [3]. Therefore, the aim of the study was to explore the support needed for sustainable lifestyle changes due to obesity among persons in the third age. The study follows the FormIT process, and this paper describes the first step; the conceptual design [4].

## 2. Background

Obesity is more common among the baby boom generation (born between 1946 and 1965) compared with those born in prior decades [5,6]. In Sweden, the prevalence of obesity (body mass index (BMI) ≥ 30) in the third age has increased in the past decade more so than among younger adults [7]. The increasing life expectancy in most countries continues to grow and the ageing population in the EU will rise from 17.1% in 2008 to 25.4% in 2035 and to 30% by 2060 [8]. As a result, we can expect health consequences in an ageing population. A Swedish national study of ageing and care (SNAC) shows that people in third age (characterized by good health, independence, and social engagement) were in overall good health and had few functional limitations [9]. The challenge of an increasing life expectancy is to remain healthy and to extend the third age, so that the fourth age (the remainder of the lifespan), which is characterised by frailty, functional loss, and cognitive decline, becomes shorter [10,11].

To extend the third age is not only about health promotion to avoid obesity, but it is also about the ability to find support for a sustainable lifestyle change. There are limitations in weight loss maintenance. Different current methods, such as diet and physical activity and cognitive behavioural techniques, are commonly used for the purpose of supporting weight loss. However, these methods have limitations in a longer perspective [12]. Studies have shown that weight loss after current methods succeeded no longer than two years [13]. A five-year follow-up study where interdisciplinary cognitive behavioural techniques, restrictions in nutrition, daily physical exercise, and relaxation sessions where used, found that 45% of individuals had either lost weight or maintained their weight. The remainder of the individuals (55%) had regained their weight or even weighed more than at their starting point [14]. Another five-year follow-up study with persons in the third age found that a new psycho-pedagogic approach (self-control, cognitive restructuring reinforcement, and relapse prevention) was successful; 70% of the patients lost weight or maintained their weight loss [15]. Despite different combined methods, reliance on physical activity, diet and/or cognitive behaviour treatment, the weight regain seems to be inevitable [16,17].

eHealth applications have been developed to deliver support for health issues such as obesity [18,19,20,21]. It is not possible to determine the effectiveness of web-based interventions in achieving or maintaining weight loss due to the heterogeneity of designs and the small number of comparable studies [19]. It is not clear what should be best practice since the technology used in the studies is rarely provided publicly [22]. eHealth based supports for lifestyle change in obesity are designed as current methods, with a focus on diet and physical activity measuring. Behavioural strategies that improve motivation, reduce stress, and assist problem-solving are missing [18]. A systematic review and meta-analysis found that mobile devices induce weight loss relative to one’s baseline and in comparison with the control group with non-intervention. The mobile device group favoured weight loss [23]. In systematic reviews of clinical trials, Allen et al. [24] found that fewer of the technology-assisted weight loss studies reported long term outcomes. There are few studies showing mobile phones used for health intervention that target older adults [25]. Delivery of health interventions through eHealth applications has the potential to be attractive to older adults. In an earlier study, we found that despite the low degree of usage in relation to healthcare, persons in the third age with self-reported overweight problems used the Internet for health issues to a greater extent than others [26]. Further, they self-rated a positive psychosocial impact on web-based eHealth services and mobile health applications. Regardless of whether the supports are with or without eHealth applications, the results are unclear and based on the same conceptual design; diet, exercise, cognitive behaviour theory, and education, and with a pedagogic approach. As these designs seem to fail in supporting a sustainable lifestyle change we need to know more about how this could be improved. Studies have shown that eHealth has the potential to support weight loss, but we need to know how the changes in lifestyle can be sustainable. Therefore, the aim of the study was to explore the support needed for sustainable changes due obesity among persons in the third age.

## 3. Materials and Methods

### 3.1. Participants

The participants in this study were recruited by healthcare professionals in a healthcare centre in Northern Sweden. To meet the aim of the study the participants must have experienced an on-going lifestyle change due to obesity, and be 55 years or older. The purposeful sample of participants were recommended to change their lifestyle due to obesity and were supported by healthcare professionals. The recruiting healthcare professionals provided the participants with verbal and written information about the study. Those who were interested replied to the first author by providing contact information. The first author contacted them and informed them again verbally about the study. Six participants, three women and three men, gave their consent to participate. One man and two women were retired and the three other participants were still working. The participants had varied experiences of using Internet and mobile health applications in relation to their health issues. All of the participants were obese and had experiences of repeated lifestyle changes due to obesity throughout their lives (see characteristics of the participants, Table 1). The study was approved by the Regional Ethical Board in Umeå, dnr (2010-293-31M, 2013-174-32M).

### 3.2. Design and Data Collection

A qualitative method was chosen, because our focus was on obtaining knowledge on beliefs and experiences of older people with obesity. The qualitative method emphasizes social interaction and human behaviour [27]. A participatory and appreciative action and reflection (PAAR) approach was used [28]. An appreciative inquiry means that instead of looking at the problems to be solved, we focused on what the participants desired (i.e., what strengths and successes we could build on) [29]. By using multistage focus group interviews, we applied a cooperative inquiry perspective whereby older persons were the co-researchers [30]. The multistage interviews were conducted with the same participants exploring a focused phenomenon in depth through three meetings to deepen the understanding of how lifestyle changes can be supported in a long-term perspective among persons in the third age. Probes were used in this study to achieve a deeper knowledge about the need for support and what support would be useful for a sustainable lifestyle change due to obesity. Probe is based on participatory design (PD) that aims at mapping out the users’ needs and developing new solutions together with them [31]. Probes can be material for self-documentation in diaries and pictures. This approach is often used in designing technical products, and can be very open-ended, aiming at understanding the user and the phenomenon [31]. All researchers (the authors), participated in all of the three multistage focus group occasions. The multistage focus group interviews were conducted during March and April 2014 at a healthcare centre in Northern Sweden.

The first meeting started with a personal presentation of each participant. This made their cooperation visible and created a group atmosphere and increased the group cohesion as a basis for the stages in the focus group interviews [32]. Thereafter the purpose of the study was communicated, and the participants signed an informed consent to participate. To visualize different experiences of lifestyle changes due to obesity in the third age, we presented findings from an earlier study, which explicated weight loss in the third age as a lifelong process of vigilance [12]. According to Wibeck [32], stimulus material can be used to reflect on in group-discussions. The participants in the focus-group were asked to speak freely and give comments to the presented experiences from the earlier study. Further, the participants were asked about when and how they experienced support in their efforts to make lifestyle changes due to obesity. “When you look back, can you please tell about a situation when it has been easy to make a change in your lifestyle? How were your experiences? If you have had bad experiences, what could have supported you?” The participants got to know each other, and built, shared, and developed stories based on their own experiences [33]. At the end of the first meeting, the participants were given a homework assignment. They were asked to write in a personal diary two times a week using cut out pictures or drawings that showed occasions when they had obesity on their minds. They were also asked to note their accompanying feelings and how they handled the situations. A diary is a typical probing instrument that focuses on routines and feelings, and it offers the added advantage of being free [34].

At the second meeting the participants were asked to share the reflections in the group about what they had written and drawn in their respective diaries. The first author presented pictures of stimulus material that symbolised free wishes and desires as a way to give an example of how to feel free to reflect without limitations. The participants chose their own special “amulet”, among different key holders, to use during coming weeks when they needed support in different situations in relation to their lifestyle changes. They were asked to reflect upon and write down in what situations they used the amulet and what function the amulet had served. In addition, they were asked to continue writing in their diaries.

At the third meeting, the interviews were based on the experiences of the amulet and the diary. The participants told about when, why, and how they had used the amulet, and how it had functioned. They talked about the content in their diaries. The participants were then asked to freely write down their wishes for support and to draw or use newspaper cut-outs (collage technique) to explain the support they wanted for a sustainable lifestyle change due to obesity [31].

All three meetings were tape recorded and directly following each multistage focus group interview the authors reflected on the group discussions. The reflections among the researchers were also tape recorded.

## 4. Data Analysis

The multistage-focus group interviews were transcribed verbatim. Together with diaries and the collages, this generated a large amount of data. The text from the diaries and the participants’ presentations of the collages were also recorded and transcribed and included in the analysis. For analysis of the data, a qualitative thematic content analysis was used. [35,36]. The data (transcriptions, diaries, and collages) was reflected upon and read several times in order to obtain an overall picture of the content and to identify patterns. This overall picture was then discussed among the authors to get a sense of the over-arching themes, trends, and patterns [37]. The next step of the analysis started with identifying meaning units of the text in relation to the purpose of the study. The meaning units (words, sentences, and paragraphs) were condensed using a description close to the text and depicting the content. Thereafter the meaning unit was condensed and coded. All coded text was then abstracted and sorted into themes and sub-themes (for examples of codes, a sub-theme, and a theme, see Table 2) When uncertainties occurred in the labelling, the original transcripts were reread and the diaries and collages were used to confirm the analysis together with the reflections made by the authors after the sessions. The sub-theme and three main themes were identified by reflections of the first and last author, where the feeling for the words in the interviews was discussed [38].

## 5. Results

In the analyses three themes were identified (Table 3). A converted way of thinking revealed a need for a changed focus from weight loss to a focus on health and activity and avoidance and reminders were needed. Managing vulnerability revealed temptations from an environment and social context that were difficult to resist and a sense of abandonment and marginalisation by healthcare. “Achieving emotional balance” revealed experiences of being steered by emotions and feeling vulnerable to others’ attitudes. The participants needed to find enjoyable and solid motivators.

### 5.1. Theme: A Converted Way of Thinking

The theme, “a converted way of thinking”, was constituted from three subthemes: “being aware of the invisible”, “needing diversion and reminders”, and “focus on health instead on weight”. The theme revealed that the participants’ experiences demonstrated a need for support to make their invisible thoughts visible. They needed support through diversion and reminders. On the other hand, they pointed out that they would be more motivated if the focus were more on the advantages of being physically active than on weight.

#### 5.1.1. Being Aware of the Invisible

In the subtheme “being aware of the invisible”, the participants expressed invisibility events and behaviours that were common to their daily living. In different situations they reacted automatically and unreflective of their situations, and it was after an occurrence that they reacted, but then it was too late. It was about discovering an awareness of their extreme eating behaviour. They found themselves standing in the kitchen and eating food, even if they were not aware of doing this. They talked about situations in which they baked a cake and then cut the cake into pieces and ate it. This eating behaviour was not about hunger; rather, it was an automatic response to food. Further, they thought about themselves in a way in which their brains were separated from the rest of the body. A support that works as a co-actor with them and not against them would be necessary: This is illustrated by the following quotation:

“At night when I wake up with terrible cravings for sweets, I have found myself sitting and eating a whole frozen cake from the freezer.”

#### 5.1.2. Needing Diversion and Reminders

The sub-theme “needing diversion and reminders” points out that when they have been conscious of their invisible actions, they needed to transform this awareness to diversion and reminders. They talked about working with perseverance and related this to earlier positive changes in their lives. Affirmative experiences need to be transformed to changes, but patience and change takes time. Situations suddenly appeared and avoidance was seen as a way to handle these unpleasant situations. This is exemplified by the following quotation:

“You know, it is like quitting smoking… When I walk behind someone who is smoking and I smell the cigarettes, and then the cravings come. You would die for a smoke, but it is only for a second and after a minute, the craving is over. And I think that it works in the same way with the appetite for sweets. If you just wait, the craving will leave you.”

The participants indicated that a diversion would be helpful even if it was invisible. It could be a cognitive diversion that hindered an automatic response and deflected actions in situations where they needed to be aware of their own behaviour. The participants talked about the amulet as a diverting tool that accompanied them in situations that were challenging, even if they could not see or feel the amulet. An amulet hindered them from the negative eating behaviour; just a feeling or the presence of the amulet stimulated them to choose alternatives that supported them to stick with their changes.

“I have thought about the amulet when I have been shopping, and at the chocolate counter, and I did not bring the amulet with me. …oh now I feel its presence, and then I left the chocolate counter. I actually acted really good.”

Other persons with obesity could function as a cautionary example. The participants stated that they felt a distance between themselves and another obese person’s behaviour. They wondered why others “like them” ate unhealthy food at cafes and restaurant and why others bought unhealthy foods in the store. TV programs about persons with obesity affected the participants as a cautionary example and reminded them of themselves. Others’ behaviour was valued, and the participants realized that these were only a way to bask in the glory of other persons with obesity. This is illustrated by the following quotation:

“I am a strange person… I’m an overweight person, and, at the same time, I can be annoyed at another overweight person who is eating a Danish pastry. I’m thinking you should not eat that because you are too overweight for that.”

The participants expressed that identification with others in the same situation was an important support in fulfilling their lifestyle changes. It gave a sense of belonging and feelings of not being isolated in difficult situations. They could share their deepest thoughts with others on a private level. As older people, it was extremely important to have someone who supported their lifestyle changes. Others’ stories fostered them to continue with the lifestyle changes, and they also found satisfaction in sharing their stories with others. Others’ stories were similar to theirs as illustrated in the following statement.

“For me it is about listening to others’ stories, and then sit right there, or I’m sitting here and thinking… so there it is. For me it's a way to sit and share with them and talk it out. For me there is nothing better than to see how I was fooling myself all the time. I think it is all about me.”

#### 5.1.3. Focus on Health Instead of Weight

In the third subtheme, “focus on health instead of weight”, the participants expressed that their life has been fraught with struggling with their changes. They were exhausted over their weight-centred identity, but even when they were absorbed with changes, it was necessary to find comfort in their attempt to find sustainable changes; otherwise life would be void of happiness and joy. They needed to stop criticising themselves and instead focus on activities that provide energy, and on a body that functions as a pleasant instrument. Their obesity hindered the participants in using their own body for things that they needed and wanted to do. Physical activities were pleasant and enjoyable, but it was difficult with a heavy body; it was sometimes painful. It was heavy to walk and to do exercises on a higher level than slow walking. This was illustrated in the following quotations:

“It is no point. I am totally confused when I think about my weight all the time… what I should eat or not. These years I still have left… I don’t care about my weight anymore.”

“I have a dog that I will take up after and I hardly can pick up after him… I am thinking about getting me a helper… I am so sick of myself.”

### 5.2. Theme: Managing Vulnerability

The theme, “managing vulnerability” consisted of three sub-themes: “coping in a social context impossible to fend off”, “handling irresistible environmental influences”, and “being confirmed”. The theme revealed that they were constantly exposed to social and cultural pressures and a surrounding environment that was a challenge to their attempt to find permanent changes. They had difficulties choosing their own paths. The participants expressed feelings of being marginalised by healthcare professionals, in that they felt that long-term support was not prioritised; there was no one to talk with about how to maintain the changes.

#### 5.2.1. Coping in a Social Context Impossible to Fend Off

In the subtheme “coping in a social context impossible to fend off” the participants expressed their thoughts regarding the cultural context and social pressures and how it is essential to leave these in order to complete their lifestyle changes. They described being fostered at a time when they could not leave food on the plate for fear of being regarded as ungrateful. There were no food alternatives; it was necessary to eat what all others ate. These were the social norms from which they could not deviate. The following statement describes this experience:

“I began to gain weight again after five years but… It was the scooter-trips, I was bullied if I had healthy food with me. It was fried pork and stuff like that everyone else was eating.”

#### 5.2.2. Handling Irresistible Environmental Influences

In the second subtheme, “handling irresistible environmental influences” indicated that it was inevitable not to be confronted and affected by the environment. Media had an impact on their eating, because it exposed food, what to eat and drink and how to look. Life was surrounded by ambient conditions that were penetrable and impossible to resist. Long ago they ate when they were hungry. In the present they expressed that they experienced continual exposure to food. Food has a central place, 24 h a day, and in every place. In the supermarket, they could both see and smell the hot dogs and fresh baked bread. Cultural events often involved eating. This is illustrated in the following statement.

“There are so terribly many diet programs on television where they are killing themselves… or it’s cooking programs… I can even sense the smells. First they are on a diet and later they bake cakes.”

#### 5.2.3. Being Confirmed

The third subtheme, “being confirmed”, expressed that the participants in the focus group had resigned themselves to the fact that they stood without support from healthcare professionals. Only routine advice had been given to them, and this advice was experienced as the same as always. Nothing really changed as they aged. Additionally, the participants experienced a sense of being marginalised; healthcare professionals had given up on them. The following statement describes this feeling.

“I sought help and there was no healthcare that was able to help with that… it is zero and nothing. They wanted to give me medicine to take (the weight) away, and that’s not my thing. It does not remove the problem; you just shut down the whole thing for a while.”

### 5.3. Theme: Achieving Emotional Balance

“Achieving emotional balance” consisted of three subthemes: “regulating feelings”, “dealing with attitudes”, and “identifying a solid motivator”. The theme revealed that the participants were steered by feelings, both sadness and happiness. Being obese was experienced as being sensitive to others’ attitudes, and the participants expressed that their motivation was often ruled by something that supported them in enjoyable, solid, and personal ways.

#### 5.3.1. Regulating Feelings

In the subtheme, “regulating feelings”, the participants stated that their life itself was controlled by feelings of sadness or happiness. They expressed that most of their feelings were comforted with an uncontrolled activity of eating. When an event occurred they responded with emotional eating and afterward, they were filled with self-loathing. It was not about having character, it was about emotions. This is illustrated by the following quotation:

“It is about very deep feelings, all the emotions that you do not put names on… self-blaming, lying… For me, it took a year to learn how to handle my feelings and realize that they exist, it takes energy to resist those traps that lurk at me, and it gives a tremendous power when you have resisted those traps.”

#### 5.3.2. Dealing with Attitudes

In the subtheme, “dealing with attitudes”, the participants expressed that others’ mindsets impacted their feelings about themselves. Other persons’ attitudes make them feel transparent. In a social context they were imperceptible; no one noticed them or listened to them. The participants also reflected that obesity had an inhibitory and reducing impact on them. They wanted to hide and felt uncomfortable with others. This was illustrated in the following statements:

“I was treated in a completely different way when I weighed 140 than when I weighed 75 kg ... in a completely different way, and it’s scary because I am the same. When I gained weight, I was thinking that they heard me when I was a normal weight. It’s scary. It is terrible that it matters so much. If you are beautiful, everyone listens to you and pays attention to what you are saying.”

“As an overweight person I reduce myself in a spiritual way.”

#### 5.3.3. Identifying a Solid Motivator

In the last subtheme, “identifying a solid motivator” it was expressed as being important to find further motivation to be able to find a sustainable change. The participants were fed up with the constant thoughts about their lifestyle changes and were not inspired to change, and this led to a sense of being abandoned and powerless. They despised themselves for not being capable of changing their own situations. They spoke of finding something in life that supported them in an individual, enjoyable way. It was necessary to find ways to reward themselves when they succeeded in their attempt to change their lifestyle and counteract the feelings of frustration and self-blaming when they had failed. They needed the ability to cope and manage change. This was expressed by the following statement:

“I changed for my own sake. That is what motivation is based on, not that I compare myself with others.”

## 6. Discussion

The aim of this study was to explore the support needed for sustainable lifestyle changes due to obesity among persons in the third age. In summary, we found that persons with obesity in the third age experienced the need to convert their way of thinking manage vulnerability, and achieve emotional balance. The converted way of thinking means that instead of focusing on weight loss, the emphasis should be on health and opportunities. Strengthening the personal capacity and self-esteem seems to be the key strategy in most health theories for behavioural changes (e.g., the transtheoretical model, the health belief model, social cognitive theory, and theories of reasoned action and planned behaviour). These health-promoting concepts emphasize individual characteristics to explain, predict, and change health behaviour, but place less stress on how to support sustainable changes (i.e., lifestyle changes). The findings in this study show that weight loss is not the principal aim, but a desirable outcome of hopefulness and personal awareness (cf. [39]). A self-empowerment methodology can enable people to convert their thinking. The concept of self-empowerment indicates a social process that involves mobilisation of a person’s own resources and development of a sense of meaning and coherence [40,41].

Our findings showed that there was a need for managing vulnerability in the process of a changing lifestyle. The constant exposure to temptations and social pressure was hard to handle and there is a need to find a strategy in situations where feelings of abandonment and marginalisation are present. In the self-determination theory (SDT) relatedness is an important psychological need [41]. Dickins et al. [42] described that blogging in an online fat-acceptance community functioned as a means to connect with others in an alternative dialogue about weight. This blogging helped the participants to feel more empowered and provided an increased sense of social relatedness. The participants in our study described that they felt abandoned by healthcare professionals, and were given no new advice about how to manage lifestyle changes. According to Aujoulat et al. [40], healthcare professionals, in order to be empowering, should provide information and choices in regard to treatment-related issues. They should also invite the person to relate the history of their illness in order to support them in the process of meaning-making through communication and dialogue [40]. Competence and autonomy are other needs stressed in SDT, and quality of motivation is also important [41]. Controlled motivation creates pressure and anxiety and causes a person to accept the shortest path to a goal. In contrast, autonomous motivation deals with interest and enjoyment and creates a sense of connectedness to one’s deepest values and beliefs. Autonomously motivation leads to increased creativity, and this has an impact on physical and social health [41].

Research shows that emotions are related to eating behaviour and obesity [43,44]. In a recent study we found that mood affects the process of weight loss [12]. In this study the participants expressed that to change their lifestyles, they needed emotional balance to find sustainability in their changes. They were overwhelmed with feelings that seemed to have control over their lives and guided their eating behaviour. This is confirmed by Zeek et al. [45] who showed that typical emotions have an impact on the intensity of the desire to eat among obese persons. The most common emotions were anger, helplessness, hurt, boredom, loneliness, powerlessness, and longing [45]. Support that helps to regulate feelings and emotions is needed. No studies in which mobile health applications served as a tool for emotional regulation and support have been found, although research shows that e-communities, social groups interacting on-line, have a tendency to increase emotions in a collective emotional state [46]. This shows that the Internet can create and modulate collective emotional expressiveness.

Weight loss mobile applications have the potential to be a helpful tool, but only a small number of behavioural strategies were included in evidence-based weight loss interventions. The majority of the apps included goal setting for weight loss and diet, and only 20% off the applications gave the user a specific physical activity goal in terms of days or minutes per week [18]. The application focus seemed to be on controlled motivation [42]. From the perspective of people in this study, they needed support for a converted way of thinking and to manage vulnerability and achieve emotional balance. This indicates that support is needed to achieve an autonomous motivation and self-empowerment for a sustainable lifestyle change.

## 7. Methodological Considerations

Trustworthiness is important in qualitative research [27]. To meet the criteria we planned three stages of focus group discussions and followed a structure for each meeting. The three authors have experience in different fields of research, which enriched the collection of data [47]. All authors participated in each multistage focus group interview session and discussed the content of each of the performed interviews, and these were also tape recorded. Codes, sub-themes, and themes were created and discussed among two of the authors several times in order to avoid bias of pre-understanding. Thereafter themes were discussed with the author from the field of innovation and design to ensure that the pre-understanding had not affected the creations of themes. We have described our research process above and have shown examples of codes that support a sub-theme and a theme (Table 2). We used quotations in the results section to strengthen the criteria of confirmability. As one of the authors was a researcher in innovation and design, we have to some extent satisfied the criterion of dependability. It might have been easier to meet the criterion of transferability if we had had more meetings. This may have revealed a greater depth in understanding the need of support. However, we do believe that the criterion for transferability was fulfilled to some extent, because the participants in this study represented persons in the third age and included both genders and a mixture of working and retired individuals. Additionally, the participants were in an ongoing lifestyle change and quest to find sustainable changes. This strengthened the variety of experiences [48].

## 8. Conclusions

In conclusion, our findings show that to achieve a sustainable lifestyle change due to obesity in the third age the focus has to be on a health identity instead of a weight identity. A personalised support with enjoyable physical activities should be designed and developed. Strategies for emotional balance based on autonomy and self-empowerment must be included, both in contact with friends as well as with health care professionals. The methodology used in this study, FormIT together with Participatory Design, whereby the focus is on the person’s perspective helped us understand their preferences and needs. This knowledge is important when new digital services and products are designed for supporting and promoting a sustainable lifestyle change in the third age. A psychosocial support seems to be an important prerequisite for sustainable change due to obesity and this could later in the process be used in an intervention program.

## Figures and Tables

**Table 1:** Characteristics of the participants.

Person	1	2	3	4	5	6
Age	61	62	68	69	73	76
Gender f/m *	m	f	m	m	f	f
Living Alone	√	√	√	√	-	√
Education Level c/u **	c	c	c	u	c	c
Health EQ5D ***	90	60	40	70	80	70
Physical Activity 30 min/daily	√	√	√	-	√	√
Used Internet for Health Information	√	√	√	-	-	√
Used Internet for Physical Activity and Exercise	√	√	√	√	-	√
Used Internet for Diet	√	√	√	-	-	√
Used Mobile Application for Diet and Weight	√	-	-	-	-	-
Used Mobile Application for Physical Activity	√	-	-	-	-	-

* f, female; m, male; ** c, college; u, university; *** EuroQol 5-dimensions of self-rated health.

**Table 2:** Example of codes, a sub-theme, and a theme.

Codes	Sub-Theme	Theme
Stranded	to Identify a Solid Motivator	Achieving Emotional Balance
Resignation	-	-
“For me”	-	-
Neutralizes	-	-
Happiness	-	-

**Table 3:** The themes with the subthemes.

Themes	A Converted Way of Thinking	Managing Vulnerability	Achieving Emotional Balance
Subthemes	Being Aware of the Invisible	Coping in a Social Context Impossible to Fend Off	Regulating Feelings
	Needing Diversion and Reminders	Handling Irresistible Environmental Influences	Dealing with Attitudes
	Focus on Health Instead of Weight	Being Confirmed	Identifying a Solid Motivator
